# Correlation of serum and local CXCL13 levels with disease severity in patients with non-traumatic osteonecrosis of femoral head

**DOI:** 10.1186/s13018-024-04645-8

**Published:** 2024-03-01

**Authors:** Yong-Heng Zhao, Wen-Xiu Zhu, Qing-He Ye, Peng Zhang, Biao-Fang Wei

**Affiliations:** 1 Guangzhou University of Traditional Chinese Medicine, Guangzhou, Guangdong Province China; 2 Department of Orthopedics, Linyi People’s Hospital, Shandong Province, China; 3 Department of Pain, Linyi People’s Hospital, Shandong Province, China

**Keywords:** Chemokine (C-X-C motif) ligand 13, Osteonecrosis of femoral head, Disease severity

## Abstract

**Objective:**

The primary aim of the present study was to explore the potential correlation of serum / local CXCL13 expressions and disease severity in non-traumatic osteonecrosis of the femoral head (NT-ONFH).

**Methods:**

In total, NT-ONFH patients (*n* = 130) together with healthy controls (HCs, *n* = 130) were included in this investigation. Radiographic progression was evaluated based on the imaging criteria outlined in the ARCO classification system. To assess the diagnostic value of serum CXCL13 in relation to radiographic progression, Receiver operating characteristic (ROC) curve analysis was conducted. Serum CXCL13 levels were quantified utilizing ELISA in all participants. Furthermore, local protein/mRNA expressions of CXCL13 were examined employing immunohistochemistry, western blot, as well as RT-PCR techniques. Clinical severity was appraised using the visual analogue scale (VAS), Harris Hip Score (HHS), and Western Ontario as well as McMaster Universities Osteoarthritis Index (WOMAC).

**Results:**

The findings revealed a significant reduction in serum CXCL13 levels among NT-ONFH patients in contrast with HCs. Moreover, both mRNA and protein expressions of CXCL13 were markedly decreased in the necrotic area (NA) than the non-necrotic area (NNA) as well as the healthy femoral head tissues. Additionally, serum CXCL13 levels were substantially lower among patients classified as ARCO stage 4 than those at ARCO stage 3. The concentrations of CXCL13 in stage 3 patients were notably diminished relative to those at ARCO stage 2. Notably, serum CXCL13 levels demonstrated a negative association with ARCO grade. Furthermore, these levels were also inversely linked to VAS scores as well as WOMAC scores while displaying a positive association with HHS scores. The findings of ROC curve suggested that reduced serum CXCL13 levels could be an underlying indicator for ARCO stage.

**Conclusions:**

The reduced levels of either serum CXCL13 or local CXCL13 were intricately linked to disease severity for patients with NT-ONFH.

**Supplementary Information:**

The online version contains supplementary material available at 10.1186/s13018-024-04645-8.

## Introduction

Osteonecrosis of the femoral head (ONFH) represents a challenging condition that can result in incapacitating hip pain and limitations on physical activity, particularly among young adults [1]. The pathophysiology of non-traumatic ONFH (NT-ONFH) is notably intricate and multifactorial, encompassing heightened bone pressure, fat embolism, microvascular injury, intravascular coagulation, and bone cell steatosis,etc., [2]. Numerous risk factors have been linked to NT-ONFH, with over 80% of cases attributed to prolonged corticosteroid use and excessive alcohol consumption [3].

Initially, the majority of ONFH patients are asymptomatic without discernible changes evident in plain radiography [4, 5], and screening for potential biomarkers conducive to early diagnosis, treatment, and prognosis stands as a reliable method [6]. The availability of a diagnostic biomarker specifically tailored for NT-ONFH could ensure prompt detection and subsequently appropriate treatment [7].

Chemokines, a family of small secretory proteins, are classified into CC, CXC, CX3C, as well as C chemokines on a basis of four conserved cysteine residues [8–10]. Known as a B-lymphocyte chemotactic, Chemokine (C-X-C motif) ligand 13 (CXCL13) is pivotal for various cellular functions, including migration, invasion, motility, proliferation, as well as apoptosis [11, 12]. CXCL13 is a mediator which is critical for either the homing or the activation of lymphoid cells [13], where its overexpression in the lymphatic region encourages the infiltration / invasion of B cells, prompting increased lymphogenesis [14]. Recent studies have underscored the influential role of CXCL13 in regulating diverse pathological processes, such as inflammatory response, cancer progression, metastasis, and drug resistance [15].

Research has indicated that CXCL13 exerts a positive reparative and promotional effect on Bone Marrow Stromal Cells (BMSCs) as well as Bone Marrow Endothelial Cells. To enhance BMSCs’ reparative effect [16], CXCL13 acts as a crucial molecular signal. In vitro chemotaxis experiments have demonstrated the chemotactic impact of CXCL13 on periosteal stromal cells. Additionally, CXCL13 along with its corresponding receptor CXCR5 are among the strongly expressed receptors in BMSCs, serving as vital chemokines governing BMSCs’ homing [17]. Another study [18] has established the close association of CXCL13 and CXCR5 with the composition and regulation of the osteogenic microenvironment in bone defects. Consequently, researchers hypothesize that the CXCR5/CXCL13 axis is pivotal for the migration process of BMSCs and constitutes an essential cellular signaling axis during bone healing. Moreover, CXCL13 has been demonstrated to promote angiogenesis [19]. Genetic knockdown of the CXCR5 receptor has markedly inhibited CXCL13-induced increases in the production as well as the angiogenesis of vascular endothelial-derived growth factor (VEGF), thereby signifying the facilitative effect of the CXCL13/CXCR5 axis on angiogenic effects [19].

While the aforementioned research illustrates the potentially protective effect of CXCL13 on NT-ONFH development, no existing studies have elucidated the plausible relationship of the levels of serum / local CXCL13 expression with the disease severity of NT-ONFH. Consequently, our study was conducted to analyze the possible correlation of the levels of serum/local CXCL13 expression and the disease severity of NT-ONFH.

## Methods

### Study subjects

Between July 2022 and July 2023, 260 individuals in total were consecutively included, involving 130 patients with a diagnosis of NT-ONFH and 130 healthy controls (HCs) from Linyi People’s Hospital, Shandong Province, China. The selection of control subjects was based on clinical symptoms, medical history, physical examination, and radiographic findings. All HCs had no osteonecrosis or other ailments. Inclusion criteria for the controls were consisted by the followings: (1) absence of prior hip pain; (2) no evidence of pelvic radiographic abnormalities; (3) absence of corticosteroid use or alcoholism history; and (4) no affiliation with the enrolled patients. Among the ONFH patients, 35 underwent total hip replacement (THR) at ARCO stage 4, while 30 patients received THR at ARCO stage 3. Additionally, 25 femoral neck fracture patients underwent THR during the same period and were enrolled as controls. Femoral head tissue samples were obtained for further analysis. All participants were Han individuals residing in or near Linyi City. Ethical approval for the research was granted by the Ethical Committee of Linyi People’s Hospital (Ethical No. 20,220,010). The study strictly adhered to the principles outlined in the Declaration of Helsinki. Informed consent was obtained from all study participants, who also completed a questionnaire providing demographic information.

### Measurement of serum CXCL13 concentration

Collection of venous blood samples (2 mL) for each participant was carried out in the morning prior to breakfast, followed by centrifugation at 3000 r/min for a duration of 5 min. The serum was then kept at the temperature of -80 °C and prepared for analysis. CXCL13’s concentration was determined utilizing an enzyme-linked immunosorbent assay (ELISA) kit.

### Real-time polymerase chain reaction (RT-PCR) for local CXCL13 mRNA expressions

Specimens of 500 mg necrotic tissue and adjacent normal tissue were obtained and immersed in 6 mL of solution at room temperature for 1 hour. Subsequently, RNA was extracted complying with the instructions provided by the manufacturer. The unpurified RNA underwent digestion with DNA enzyme at 37°C for 1 hour, followed by inactivation with DNA enzyme inactivation solution. The RNA was then extracted from the supernatant using 0.5 mol/L ammonium acetate and left overnight in -20°C with a volume fraction of 100% ethanol. The resulting particles were repositioned in 60 µL eluent and stored at -80°C. Reverse transcription of RNA into cDNA was achieved using a reverse transcription kit. The primer sequences used were as follows: CXCL13: Forward: 5’- GCTTGAGGTGTAGATGTGTCC − 3’, Reverse: 5’- CCCACGGGGCAAGATTTGAA − 3’; β-actin: Forward: 5’- TAGGCCGTGGCTCAAGAAC − 3’, Reverse: 5’- TGCATCTCCAAGTTGCCTTTG − 3’. The SYBR Green method was employed to assess the mRNA expression levels. The reaction conditions entailed pre-denaturation at 95°C for 34 s for one cycle; denaturation at 95°C for 5 s, annealing at 60°C for 34 s, followed by elongation at 72°C for 30 s, summing up to 40 cycles. Data was captured using the ABI7500 fluorescence quantitative RT-PCR instrument and computed as CT values of the relevant genes. Data analysis was conducted utilizing the 2-^△△CT^ method to ascertain the differential expression of the target gene in the experimental group in contrast with the control group.

### Western blotting for CXCL13 proteins

Sample proteins underwent separation via agarose- sodium dodecyl sulfate polyacrylamide gel electrophoresis (SDS-PAGE), followed by transfer onto polyvinylidene fluoride (PVDF) membranes. Next, the membranes were blocked using 3% bovine serum albumin (BSA) in phosphate buffered saline (PBS) and probed with CXCL13 antibody (Abcam, Cambridge, UK) or the Glyceraldehyde-3-phosphate dehydrogenase (GAPDH) antibody (Abcam, Cambridge, UK) as the primary antibody. Besides, anti-rabbit immunoglobulins (Abcam, Cambridge, UK) were utilized as horseradish peroxidase-conjugated secondary antibodies. In addition, semi-quantitative analysis of the enhanced chemiluminescence (ECL) western blot detection system was employed for the visualization of specific proteins.

### Immunohistochemistry

The femoral heads were coronally halved. A bone section measuring 1.0 × 1.0 × 0.3 cm was then obtained from the necrotic area (NA) as well as the adjacent non-NA in ONFH patients as well as from the femoral neck fracture femoral head. Specimens of the femoral head were fixed with a 10 g/L paraformaldehyde solution, decalcified using 5% Ethylenediamine tetraacetic acid (EDTA), dehydrated with gradient ethanol, embedded in paraffin, and subsequently sectioned routinely. The tissues were subjected to washing using 20% H_2_O_2_ distilled water at room temperature for 5–10 min for endogenous enzyme inactivation, repeated three times. Following this, the slices were immersed in citrate buffer (0.01 mol/L) and heated in a pressure cooker until boiling. After rinsing twice with PBS for antigen repair, the specimens were blocked using 5% BSA at 37°C for 15 min, followed by the overnight incubation at the temperature of 4°C with anti-CXCL13 IgG (1:200, Cat No. ab272874, Abcam, Cambridge, UK). The slides were subjected to an overnight incubation after triply washed by PBS. Following another wash with PBS, the slides were placed in the dark at 37˚C with a secondary antibody (1:50; R&D Systems, Inc., Minneapolis, MN, USA) for a duration of 30 min. Post 3,3’-diaminobenzidine (DAB) color rendering, we observed the sections, followed by the capture of images. The measurement of integrated optical density (IOD) values was carried out utilizing Image-Pro Plus software version 6.0 (Media Cybernetics, Inc., Rockville, MD, USA).

### Assessment of radiographic severity

The radiographic severity was assessed utilizing the 2019 Revised Association Research Circulation Osseous (ARCO) Classification System for ONFH [20]. Specifically, Stage 1 involved normal radiographs alongside abnormal MRI findings, while Stage 2 was characterized by the absence of a crescent sign, in addition to radiographic indications of sclerosis, osteolysis, or localized osteoporosis. Stage 3 encompassed subchondral fracture, necrotic segment fracture, and/or flattening of the femoral head as observed on radiographic or CT imaging. Lastly, Stage 4 involved signs of osteoarthritis, narrowing of joint spaces, and degenerative alterations in the acetabulum. Enrollment of patients took place when the ACRO grade was ≥ 2. In cases where both hips were affected, the more severe side was selected. Radiological findings were independently reviewed by two experienced radiologists, and a *Kappa* value was adopted to determine their interpretations’ consistency.

### Assessment of disease severity

The disease severity among ONFH patients was assessed utilizing the visual analogue scale (VAS), Harris Hip Score (HHS), as well as Western Ontario and McMaster Universities Osteoarthritis Index (WOMAC).

VAS score, a pain level scoring standard, is commonly employed to gauge pain severity, scored on a 0 to 10 scale, among which 0 indicates no pain while 1–3 indicates mild pain.

Harris Hip Score is widely utilized for quantitative evaluation of hip joint functional status before and after surgery, encompassing four aspects, including pain, function, deformity, as well as joint range of motion. Scores between 90 and 100 are categorized as excellent, while those falling within the range of 80 to 89 are deemed good. Scores from 70 to 79 are considered acceptable, and values equal to or less than 69 points indicate a poor condition.

WOMAC scale comprises three dimensions, including pain, stiffness, as well as physical function, comprising 24 items in total, including 5 items for pain, 2 items for stiffness, as well as 17 items for physical function, encompassing fundamental symptoms and signs of osteoarthritis. As a widely used assessment tool for specific diseases, WOMAC effectively reflects and evaluates joint pain and function improvement subsequent to joint replacement surgery, demonstrating high reliability and effectiveness in evaluating osteoarthritis patients.

### Statistical analysis

Statistical analyses were conducted utilizing SPSS 13.0. Normally distributed measurement data were expressed as mean ± standard deviation (SD). Comparisons between groups was performed via t-tests. Multiple group comparisons were conducted through analysis of variance (ANOVA) as well as SNK tests. Abnormally distributed measurement data were presented as medians (quartile). Comparison of two groups was performed utilizing Mann–Whitney-Wilcoxon test while the comparison across multiple groups was carried out utilizing the nonparametric Kruskal-Wallis test. Besides, correlation between variables were conducted utilizing Pearson or Spearman correlation analysis. Categorical data were expressed with percentages and their distribution across multiple groups was compared utilizing Chi-square tests. *P* < 0.05 indicated the statistical significance.

## Results

### Demographic data

**Fig. 1:**
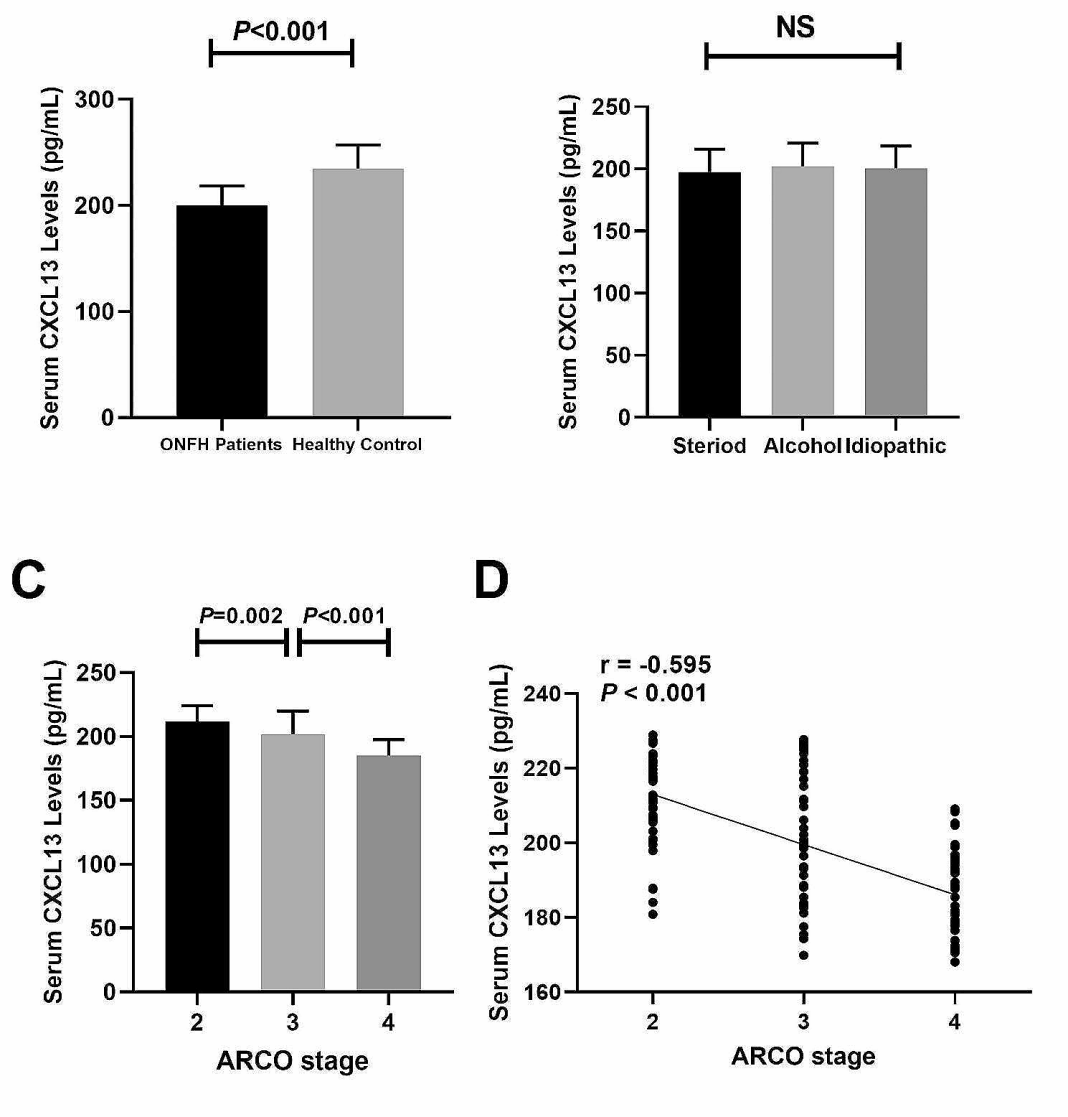
(**A**) Comparison of serum CXCL13 levels of NT-ONFH group with healthy controls (**B**) A. Comparison of serum CXCL13 levels among patients with ONFH patients induced by steroids and alcohols, and idiopathic ONFH. (**C**) Comparison of serum CXCL13 levels across distinct ARCO stages. (**D**) Association of serum CXCL13 levels and ARCO stages

The demographic characteristics of participants are listed in Table 1. Out of the 130 NT-ONFH patients, 80 were male and 50 were female, with a mean age was 47.79 ± 8.45 years as well as a mean BMI of 22.14 ± 3.15 kg/m^2^. In HC group comprising 130 HCs, 75 were male and 55 were female, with an average age of 48.23 ± 8.33 years as well as mean BMI of 22.90 ± 2.73 kg/m^2^. We didn’t observe any differences with statistical significance regarding age, sex distribution, as well as BMI when compared NT-ONFH patients to HCs (Table 1). Serum CXCL13 levels decreased with statistical significance among NT-ONFH patients (200.0 ± 18.3 pg/mL) in contrast with HCs (234.6 ± 22.3 pg/mL) (*P* < 0.001) (Fig. 1A). The study included ONFH patients induced by steroids (*n* = 48), ONFH patients induced by alcohols (*n* = 47), together with idiopathic ONFH patients (*n* = 35), with no significant differences observed among these groups, respectively (197.7 ± 18.3 pg/mL vs. 202.1 ± 18.7 pg/mL vs. 200.5 ± 18.0 pg/mL, *P* > 0.05) (Fig. 1B).

**Table 1:** Demographic features of Non-traumatic ONFH patients and healthy controls

	Non-traumatic ONFH patients (*n* = 130)	Healthy Controls (*n* = 130)	*P* value
Age(Y)	47.79 ± 8.45	48.23 ± 8.33	0.179
Gender(F/M)	50/80	55/75	0.818
BMI	22.14 ± 3.15	22.90 ± 2.73	0.314
VAS score	5.0 ± 1.9	/	
HHS score	68.0 ± 8.7	/	
WOMAC score	30.0 ± 4.7	/	
ARCO stage (2/3/4)	45/45/40	/	
Aetiology			
Steroid/Alcoholic/Idiopathic	48/47/35	/	
Serum CXCL13 Levels (pg/mL)	200.0 ± 18.3	234.6 ± 22.3	< 0.001

**Table 2:** Correlation of serum CXCL13 levels with, ARCO stage, HHS, VAS and WOMAC scores in non-traumatic ONFH patients adjusted by age and BMI

	Serum CXCL12 levels (pg/mL)		Serum CXCL12 levels (pg/mL) *	
Variables	*R*	*P*	*R’*	*P*
BMI	0.019	> 0.05	/	/
Age	0.101	> 0.05	/	/
VAS Scores	-0.622	< 0.001	-0.577	< 0.001
HSS Scores	0.565	< 0.001	0.511	< 0.001
WOMAC Scores	-0.405	< 0.001	-0.357	0.017
ARCO stage	-0.595	< 0.001	-0.551	< 0.001

*Adjusted by age and BMI

### Relationship of serum CXCL13 concentrations with radiographic severity

Serum CXCL13 levels of 130 NT-ONFH cases at distinct ARCO stages were examined. Following radiographic assessment, NT-ONFH group were categorized into 3 groups: ARCO stage 2 (*n* = 45), ARCO stage 3 (*n* = 45), and ARCO stage 4 (*n* = 40). Patients at ARCO stage 4 (185.0 ± 12.6 pg/mL) exhibited markedly decreased serum CXCL13 concentrations in contrast with those at ARCO stage 3 (201.5 ± 18.5 pg/mL) (*P* < 0.001) (Fig. 1C). Patients at ARCO stage 3 showed reduced serum CXCL13 levels (201.5 ± 18.5 pg/mL) with statistical significance in contrast with those at ARCO stage 2 (212.0 ± 12.0 pg/mL) (*P* = 0.002) (Fig. 1C). Serum CXCL13 levels demonstrated a negative association with ARCO stages (*r* = -0.595, *P* < 0.001) (Fig. 1D).

### Local CXCL13 expression in non-tranmatic ONFH femoral heads

**Fig. 2:**
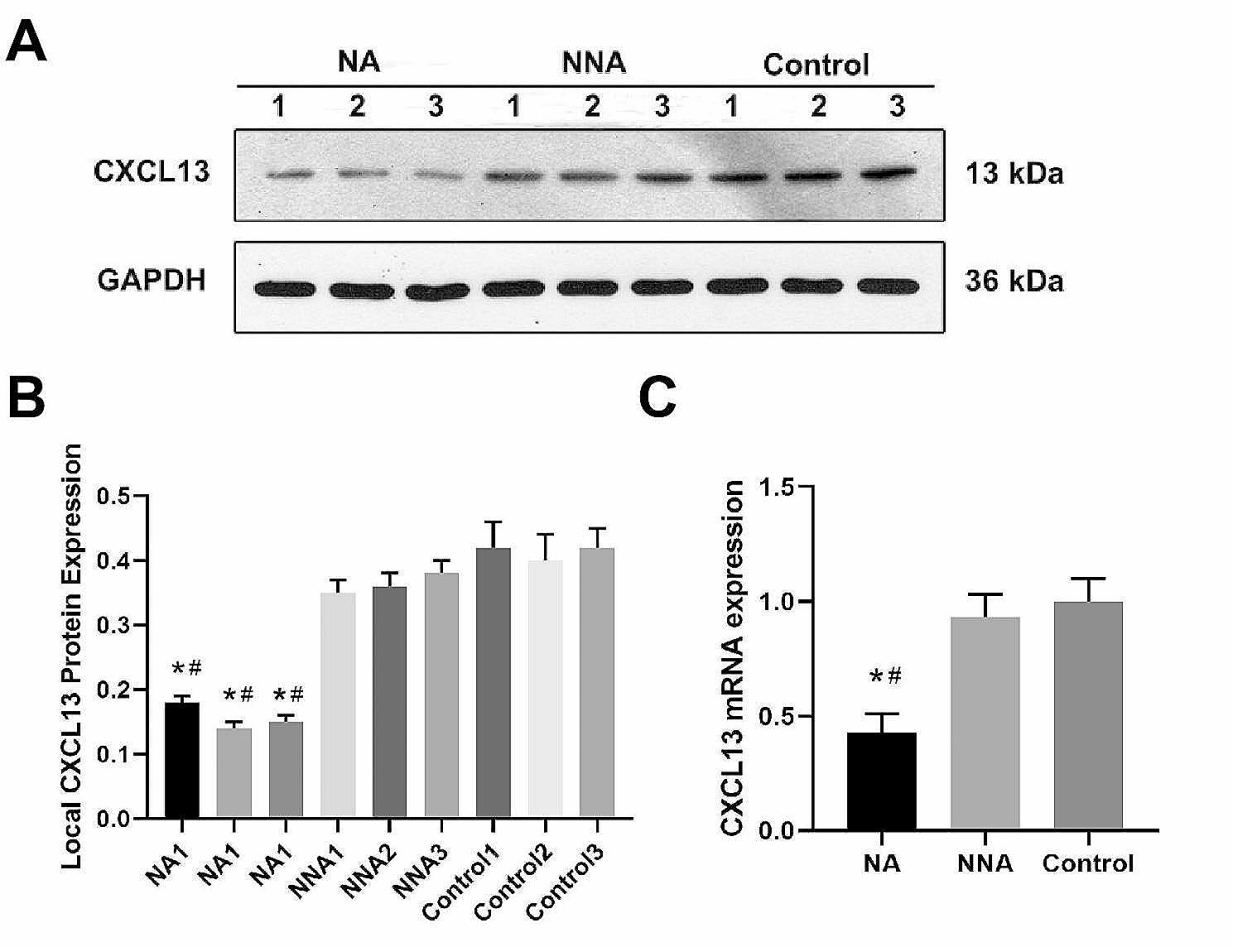
**A** Representative bands of western blot bands among NA, NNA, and controls. **B.** Quantitation of CXCL13 protein expressions among NA, NNA, and controls. **C.** Quantitation of CXCL13 mRNA expressions among NA, NNA, and controls. Data are expressed as the mean ± SD. NA: necrotic area; NNA: non-NA. *P < 0.05 vs. NA. ^#^*P* < 0.05 vs. control

**Fig. 3:**
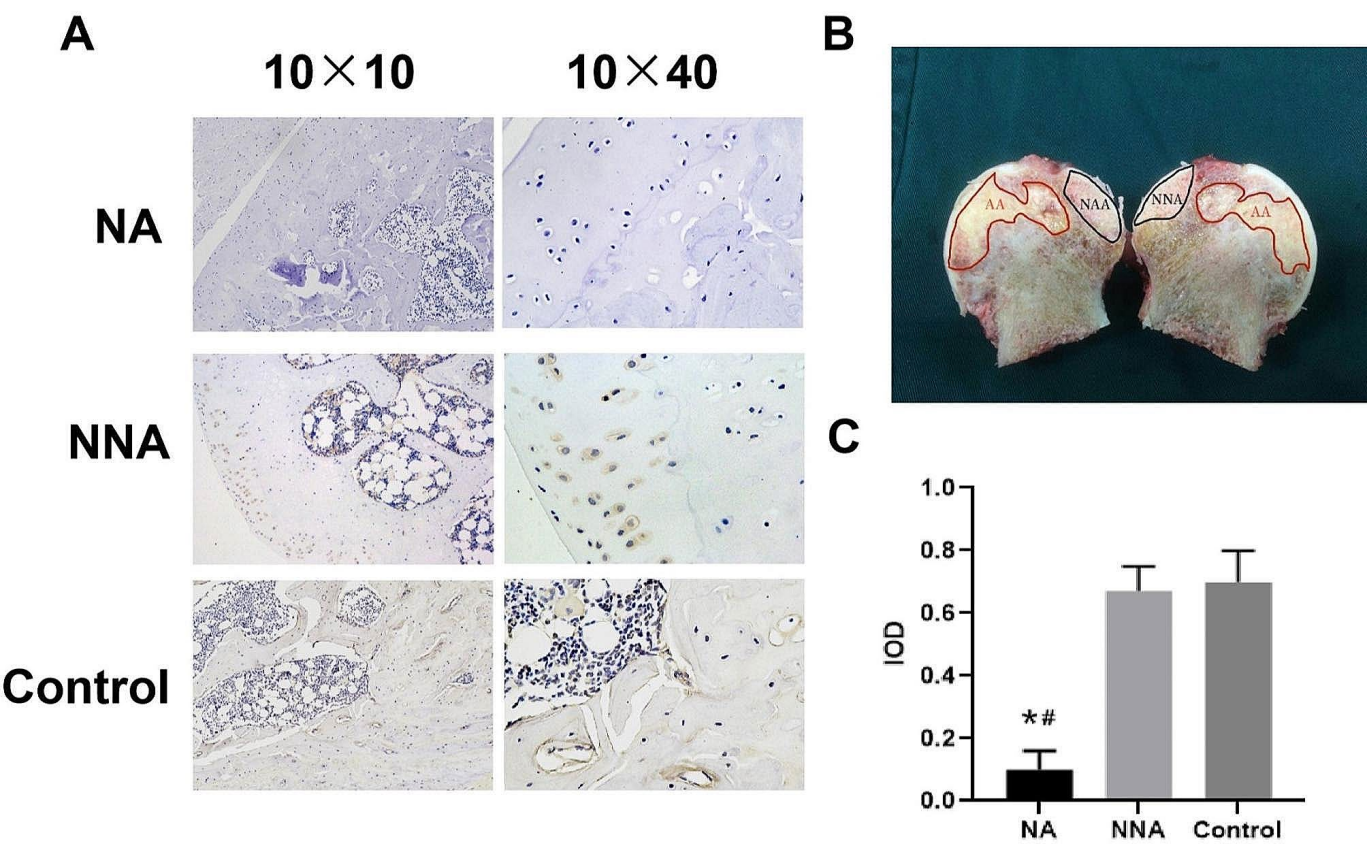
(**A**) CXCL13 immunohistochemical staining from NA, NNA, and control group at magnification 10 × 10 and 10 × 40 (**B**) Femoral head samples from ONFH patients and the NA and NNA groups were indicated (**C**) Quantified analysis of CXCL13 protein expression. IOD of CXCL13 positive cells were significantly lower in the NA group than the NNA group and controls. Data are expressed as the mean ± SD. IOD, integrated optic density; NA: necrotic area; NNA: non-NA. * *P* < 0.05 vs. NNA. ^#^*P* < 0.05 vs. controls

Local expression of CXCL13 in non-traumatic ONFH femoral heads was investigated. Both CXCL13 mRNA and protein expressions in the the femoral head’s NA were notably lower than the NNA group and the femoral neck fracture control (Fig. 2A, B and C). Immunohistochemical staining for CXCL13 protein revealed a reduction in the NA group with statistical significance in contrast with NNA / HC group (Fig. 3A, B). Image-Pro Plus software was utilized for semi-quantitative analysis of the IOD values of CXCL13 expression. Besides, the mean IOD value was 0.67 ± 0.08 for the NNA group and 0.70 ± 0.10 for the control group, while it was 0.10 ± 0.06 for the NA group (Fig. 3C), indicating obvious differences when compared the NA group to NNA and the control groups.

### Relationship of serum CXCL13 concentrations and symptomatic severity

**Fig. 4:**
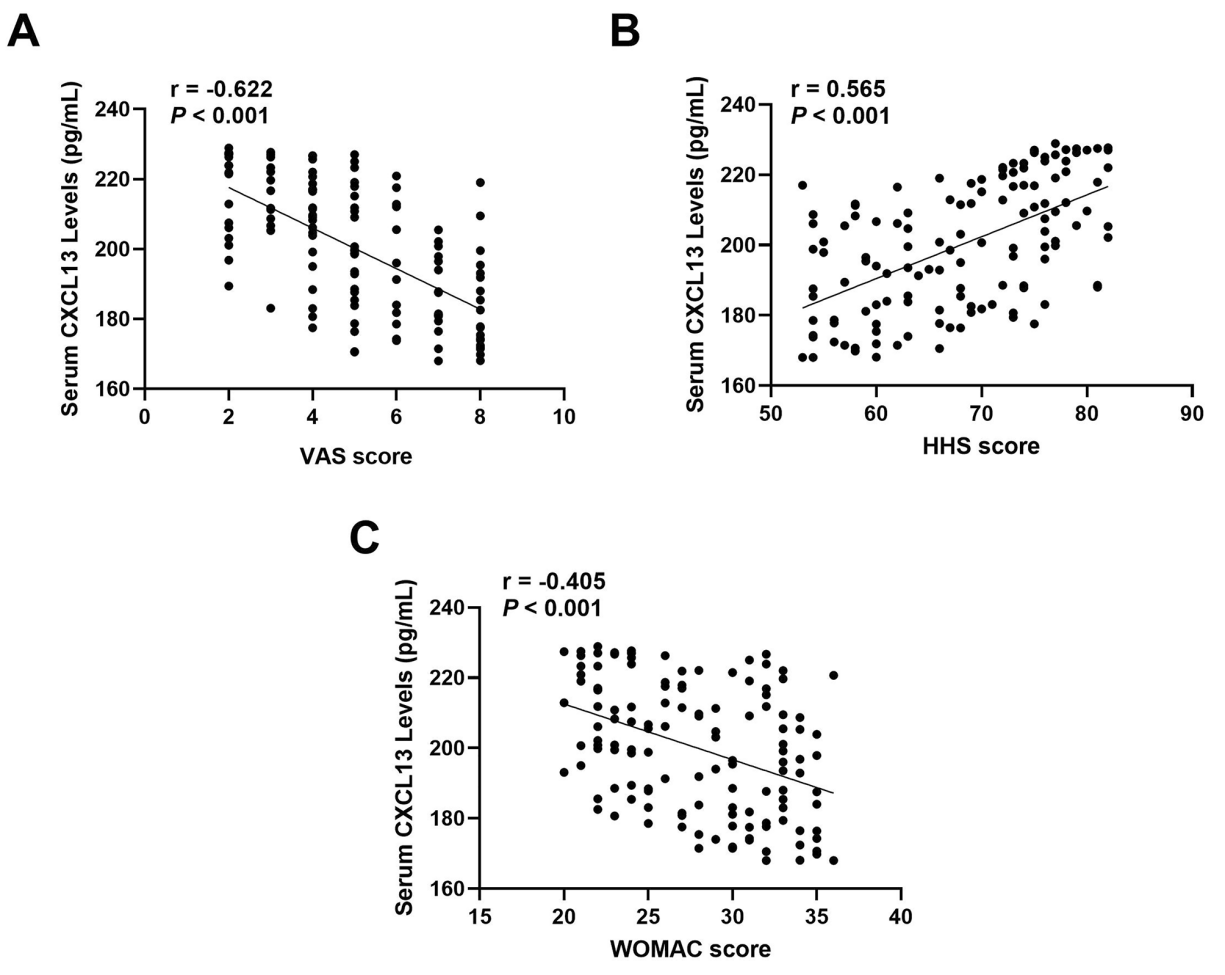
(**A**) Correlation of serum CXCL13 levels with VAS score (**B**) Correlation of CXCL13 levels with HHS score (**C**) Correlation of serum CXCL13 levels with WOMAC score

The potential correlation of serum CXCL13 levels and clinical severity utilizing VAS, WOMAC, as well as HHS scores was explored. Serum CXCL13 levels were in a negative correlation with VAS scores (*r* = -0.622, *P* < 0.001) (Fig. 4A), WOMAC scores (*r*= -0.405, *P* < 0.001) (Fig. 4C), but in a positive association with HHS (*r* = 0.565, *P* < 0.001) (Fig. 4B). These associations had statistical significance following the adjustment for both age and BMI (Table 2).

### Receiver operating characteristic (ROC) curve analysis

**Fig. 5:**
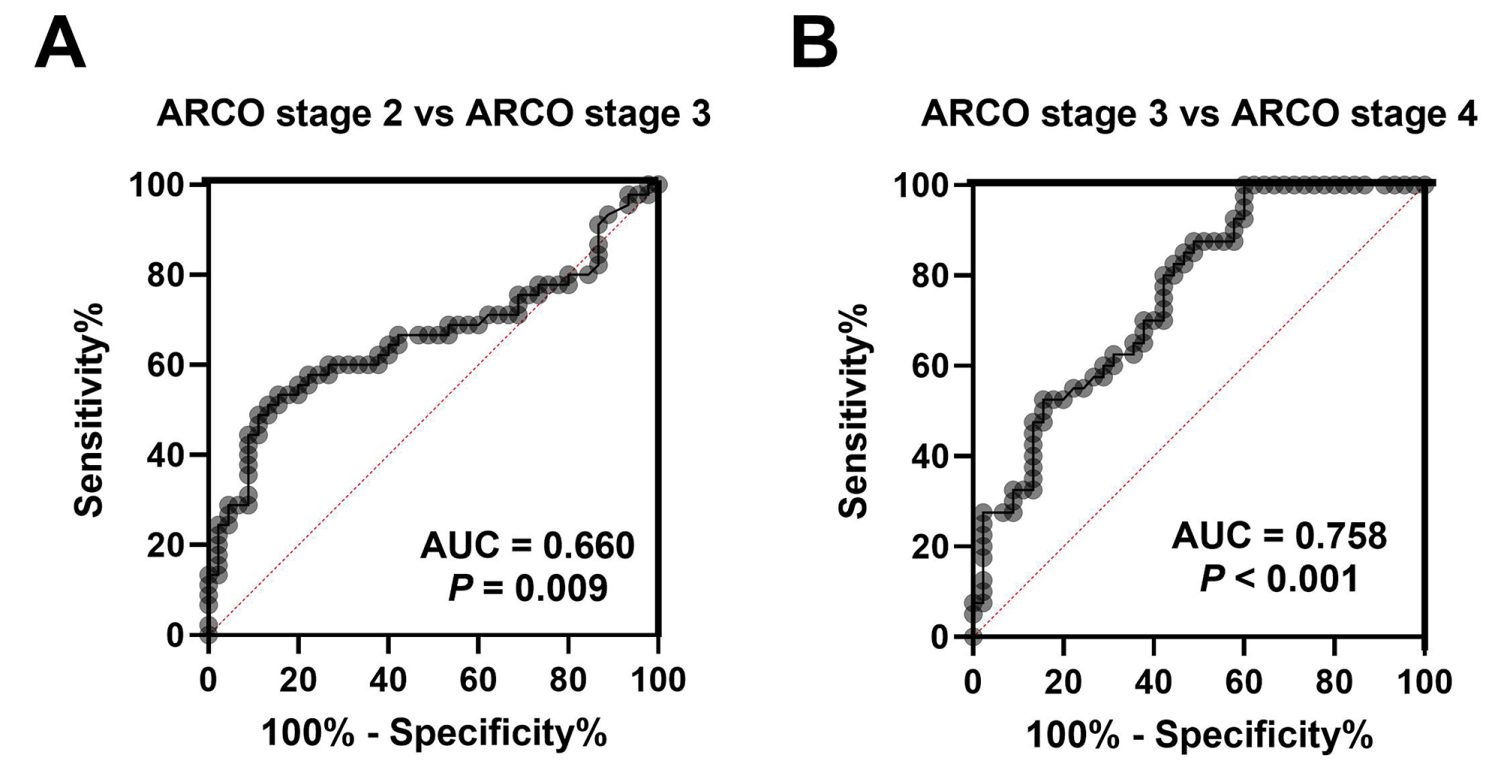
**A.** ROC curve analysis of serum CXCL13 regarding ARCO stage 2 vs. stage 3 **B.** ROC curve analysis of serum CXCL13 regarding ARCO stage 3 vs. stage 4

The assessment of the potential diagnostic value of CXCL13 concerning ARCO stage was carried out utilizing ROC curve analysis. As illustrated in Fig. 5, reduced serum CXCL13 levels exhibited high areas under the curve (AUCs) with statistical significance for both ARCO stage 2 vs. ARCO stage 3 and ARCO grade 3 vs. ARCO grade 4 (ARCO stage 2 vs. ARCO stage 3: AUC = 0.660, *P* = 0.009; ARCO stage 3 vs. ARCO stage 4: AUC = 0.758, *P* < 0.001) (Fig. 5A, B). These results suggest the decreased serum CXCL13 to be a valuable diagnostic biomarker for NT-ONFH at various stages of the condition.

## Discussion

This study analyzed the local / serum expressions of CXCL13 among NT-ONFH patients, and further investigated the relationship of serum CXCL13 concentrations with disease progression. To our knowledge, we present the first evidence that reduced local and serum CXCL13 levels were linked to the symptomatic / radiographic severity of NT-ONFH. These associations were still statistically significant even after the adjustment for age as well as BMI. These results suggest that CXCL13 may be crucial in protecting against ONFH and could potentially be a reliable biomarker for the disease severity for patients with NT-ONFH.

The pathological changes linked to NT-ONFH are challenging to detect early on due to the femoral head’s deep position within the muscular layer. Once the femoral head is found to be affected, its lesions often progress to bone cell necrosis, bone trabecular deformation, atrophy, or may have already undergone insect-like changes and punctate sclerosis. Therefore, it is urgent to identify potential diagnostic biomarkers.

Excessive use of hormones or alcohol weakens osteogenic differentiation and hampers the blood supply to the femoral head, ultimately contributing to local bone tissue collapse, further resulting in its necrosis. A study [21] also revealed that CXCL13 is in a close association with the composition and regulation of the osteogenic microenvironment of bone defects. CXCL13 exhibits a strong chemotactic effect on BMSCs, inducing them to migrate to the injured site, recruiting BMSCs, and reconstructing the osteogenic microenvironment, establishing osteogenic activities with BMSCs as the “chemotactic core” [22]. Additionally, CXCL13 can promote BMSCs to enhance tendon-bone healing in rats in vitro [23]. CXCL13 directly regulates cell proliferation and activity, induces bone marrow mesenchymal stem cells to migrate to the injured area, and stimulates osteoblast differentiation [24–26]. Furthermore, the CXCL13/CXCR5 axis can promote endothelial progenitor cell homing and angiogenesis [19]. Increased VEGF generation and endothelial precursor cell angiogenesis induced by CXCL13 were significantly inhibited by CXCR5 receptor gene knockout, suggesting that the CXCL13/CXCR5 axis has a potential in promoting angiogenesis among human endothelial progenitor cells.

In our study, we initially observed a reduction in both CXCL13 mRNA and protein expressions compared to controls, suggesting that CXCL13 may play a beneficial role in normal femoral head function. The local alteration of CXCL13 was consistent with its change in serum. Furthermore, decreased systemic CXCL13 levels were linked to poorer clinical outcomes, indicating a correlation between CXCL13 expression and hip function in NT-ONFH patients.

Our study possesses several limitations that warrant consideration. Firstly, this study is a single-center investigation characterized by a relatively limited sample size focused on the Chinese racial group. Further comprehensive studies involving larger, multi-center samples inclusive of various racial groups are needed to validate this study’s findings. Secondly, our study solely examined serum CXCL13, and the examination of other potential chemokines might provide more valuable insights. Thirdly, this study exclusively focused on evaluating the serum / local expression of CXCL13 among the patients with NT-ONFH, highlighting the need for additional research to explore its potential utilization in assessing therapeutic efficacy as well as underlying mechanisms of action in ONFH.

In summary, our results indicate that decreased local and serum CXCL13 expression is associated with the development as well as the severity of NT-ONFH. Additionally, CXCL13 may be a valuable tool for evaluating disease severity among patients with NT-ONFH.

### Electronic supplementary material

Below is the link to the electronic supplementary material.

Supplementary Material 1

## Data Availability

No datasets were generated or analysed during the current study.

## References

[1] Quaranta M, Miranda L, Oliva F (2021). Osteotomies for avascular necrosis of the femoral head. Br Med Bull.

[2] Sadile F, Bernasconi A, Russo S (2016). Core decompression versus other joint preserving treatments for osteonecrosis of the femoral head: a meta-analysis. Br Med Bull.

[3] Migliorini F, La Padula G, Oliva F (2022). Operative management of avascular necrosis of the femoral head in skeletally immature patients: a systematic review. Life (Basel).

[4] Migliorini F, Maffulli N, Baroncini A (2023). Prognostic factors in the management of osteonecrosis of the femoral head: a systematic review. Surgeon.

[5] Migliorini F, Maffulli N, Eschweiler J (2021). Core decompression isolated or combined with bone marrow-derived cell therapies for femoral head osteonecrosis. Expert Opin Biol Ther.

[6] Wang XY, Hua BX, Jiang C (2019). Serum biomarkers related to glucocorticoid-Induced osteonecrosis of the femoral head: a prospective nested case-control study. J Orthop Res.

[7] Floerkemeier T, Budde S, Willbold E (2021). Do biomarkers allow a differentiation between osteonecrosis of the femoral head and osteoarthritis of the hip? - a biochemical, histological and gene expression analysis. Osteoarthritis Cartilage.

[8] Legler DF, Thelen M. New insights in chemokine signaling.F1000Res. 2018;7:95. 10.12688/f1000research.13130.1 PMC5782407 29416853

[9] Miller MC, Mayo KH (2017). Chemokines from a structural perspective. Int J Mol Sci.

[10] Beck-Sickinger AG, Panitz N (2014). Semi-synthesis of chemokines. Curr Opin Chem Biol.

[11] Finch DK, Ettinger R, Karnell JL (2013). Effects of CXCL13 inhibition on lymphoid follicles in models of autoimmune disease[J]. Eur J Clin Investig.

[12] Hussain M, Adah D, Tariq M (2019). CXCL13/CXCR5 signaling axis in cancer[J]. Life Sci.

[13] Armas-González E, Domínguez-Luis MJ, Díaz-Martín A (2018). Role of CXCL13 and CCL20 in the recruitment of B cells to inflammatory foci in chronic arthritis[J]. Arthritis Res Ther.

[14] Humby F, Bombardieri M, Manzo A (2009). Ectopic lymphoid structures support ongoing production of class-switched autoantibodies in rheumatoid synovium[J]. PLoS Med.

[15] Yoshitomi H (2020). CXCL13-producing PD-1(hi)CXCR5(-) helper T cells in chronic inflammation[J]. Immunol Med.

[16] Jiang H, Wang Y, Meng J (2018). Effects of transplanting bone marrow stromal cells transfected with CXCL13 on Fracture Healing of Diabetic rats. Cell Physiol Biochem.

[17] Lisignoli G, Toneguzzi S, Grassi F (2002). Different chemokines are expressed in human arthritic bone biopsies: IFN-gamma and IL-6 differently modulate IL-8, MCP-1 and rantes production by arthritic osteoblasts[J]. Cytokine.

[18] Smith H, Whittall C, Weksler B et al. Chemokines stimulate bidirectional migration of human mesenchymal stem cells across bone marrow endothelial cells[J]. Stem Cells Dev 2012,21(3):476–86. 10.1089/scd.2011.0025 21513440

[19] Tsai CH, Chen CJ, Gong CL (2021). CXCL13/CXCR5 axis facilitates endothelial progenitor cell homing and angiogenesis during rheumatoid arthritis progression[J]. Cell Death Dis.

[20] Yoon BH, Mont MA, Koo KH (2020). The 2019 revised version of Association Research Circulation Osseous staging system of osteonecrosis of the femoral head. J Arthroplasty.

[21] Pietrasanta C, De Leo P, Jofra T (2021). CXCR5-CXCL13 axis markers in full-term and preterm human neonates in the first weeks of life. Eur J Immunol.

[22] Zeng J, Xiong S, Ding L (2019). Study of bone repair mediated by recombination BMP-2/ recombination CXC chemokine ligand-13-loaded hollow hydroxyapatite microspheres/chitosan composite. Life Sci.

[23] Tian F, Ji XL, Xiao WA (2015). CXCL13 promotes the effect of bone marrow mesenchymal stem cells (MSCs) on tendon-bone healing in rats and in C3HIOT1/2 cells. Int J Mol Sci.

[24] Lisignoli G, Toneguzzi S, Piacentini A (2006). CXCL12 (SDF-1) and CXCL13 (BCA-1) chemokines significantly induce proliferation and collagen type I expression in osteoblasts from osteoarthritis patients. J Cell Physiol.

[25] Cristino S, Piacentini A, Manferdini C (2008). Expression of CXC chemokines and their receptors is modulated during chondrogenic differentiation of human mesenchymal stem cells grown in three-dimensional scaffold: evidence in native cartilage. Tissue Eng Part A.

[26] Tian F, Ji XL, Xiao WA (2015). CXCL13 promotes osteogenic differentiation of mesenchymal stem cells by inhibiting miR-23a expression. Stem Cells Int.

