# Supplementary material for: Correlation of serum and local CXCL13 levels with disease severity in patients with non-traumatic osteonecrosis of femoral head
Source: J Orthop Surg Res. 2024 Mar 1;19:162. doi: 10.1186/s13018-024-04645-8 (PMC10908116; doi:10.1186/s13018-024-04645-8)
Supplement: Supplementary file 1 — Supplementary Material 1 [file 13018_2024_4645_MOESM1_ESM.docx]

**Clinical significance**

1.Serum CXCL13 levels in non-traumatic osteonecrosis of femoral head patients are significantly reduced in contrast with healthy controls.

2.Both mRNA and protein expressions of CXCL13 were markedly decreased in the necrotic area than the non-necrotic area as well as the healthy femoral head tissues.

3.The reduced levels of either serum CXCL13 or local CXCL13 were intricately linked to disease severity for patients with non-traumatic osteonecrosis of femoral head.
